# Impact of CyberKnife Radiosurgery on Overall Survival and Various Parameters of Patients with 1-3 versus ≥ 4 Brain Metastases

**DOI:** 10.7759/cureus.1798

**Published:** 2017-10-24

**Authors:** Judith Murovic, Victoria Ding, Summer S Han, John R. Adler, Steven D. Chang

**Affiliations:** 1 Department of Neurosurgery, Stanford University School of Medicine; 2 Quantitative Sciences Unit, Department of Medicine, Stanford University School of Medicine; 3 Quantitative Sciences Unit, Department of Medicine, Stanford University School of Medicine

**Keywords:** 1 to 3 and 4 and more brain metastases, cyberknife radiosurgery, brain metastases, overall survival, absolute lymphocyte count, absolute neutrophil count, nlr

## Abstract

Introduction

This study’s objective is to compare the overall survivals (OSs) and various parameters of patients with 1-3 versus ≥ 4 brain metastases post-CyberKnife radiosurgery (CKRS) (Accuray, Sunnyvale, California) alone.

Methods

Charts of 150 patients, from 2009-2014, treated with only CKRS for brain metastases were reviewed retrospectively for overall survival (OS) and patient, tumor, and imaging characteristics. Parameters included demographics, Eastern Cooperative Oncology Group (ECOG) performance scores, number and control of extracranial disease (ECD) sites, cause of death (COD), histology, tumor volume (TV), and post-CKRS whole brain radiotherapy (WBRT). The imaging characteristics assessed were time of complete response (CR), partial response (PR), stable imaging or local failure (LF), and distal brain failure (DBF). Patients and their data were divided into those with 1-3 (group 1) versus ≥ 4 brain metastases (group 2). For each CR and LF patient, absolute neutrophil count (ANC), absolute lymphocyte count (ALC)), and ANC/ALC ratio (NLR) were obtained, when available, at the time of CKRS.

Results

Both group 1 and group 2 had a median OS of 13 months. The patient median age for the 115 group 1 patients versus the 35 group 2 patients was 62 versus 56 years. Group 1 had slightly more males and group 2, females. The predominant ECOG score for each group was 1 and the number of ECD sites was one and two, respectively. Uncontrolled ECD occurred in the majority of both group 1 and group 2 patients. The main COD was ECD in both groups. The prevalent tumor histology for groups 1 and 2 was non-small cell lung carcinoma. Median TVs were 1.08 cc versus 1.42 cc for groups 1 and 2, respectively. The majority of patients in both groups did not undergo post-CKRS WBRT. Imaging outcomes were LC (CR, PR, or stable imaging) in 93 (80.9%) and 26 (74.3%) group 1 and 2 patients, of whom 32 (27.8%) and six (17.1%) had CR; 38 (33.0%) and 18 (51.4%), PR and 23 (20.0%) and two (5.7%), stable imaging; LF was the outcome in 22 (19.1%) and nine (25.7%) patients, and DBF occurred in 62 (53.9%) and 21 (60.0%), respectively. Uni- and multivariable analyses showed the independent parameters of a lower ECOG score, a greater number of ECD sites and uncontrolled ECD were significantly associated with greater mortality risk with and without accounting for other covariates. At CKRS, 19 group 1 and 2 CR patients had a mean ANC of 5.88 K/µL and a mean ALC of 1.31 K/µL and 13 (68%) of 19 had NLRs ≤ five, while 11 with LFs had a mean ANC of 5.22 K/µL and a mean ALC of 0.93 K/µL and seven (64%) had NLRs > five. An NLR ≤ five and high ALC was associated with a CR and an NLR > five and a low ALC with an LF.

Conclusions

Median OS post-CKRS was 13 months for both patients with 1-3 brain metastases and with ≥ 4. This is the only study in the literature to evaluate OS in patients with 1-3 and ≥ 4 brain metastases who were treated with CKRS alone. For groups 1 and 2 patients combined, 119 (79.3%) had LC and 38 (25.3%) had CR. The ANC, ALC, and NLR values are likely predictive of CR and LF outcomes

## Introduction

Brain metastases are present at the time of diagnosis of a primary carcinoma in 20-40% of patients [1-4]. Improved overall survival (OS) is increasing the incidence of brain metastases and is due to novel chemo- and immunotherapies and better imaging techniques, enabling earlier detection of punctate and silent brain lesions [5].

Eighty percent of patients with brain metastases present with 1-3 lesions [6]. Correspondingly, Tsao et al. presented their 2010 survey results analyzing brain metastasis management: 68% of mainly radiation oncologist respondents chose 1-3 brain metastases as the ideal number to treat with stereotactic radiosurgery (SRS) alone. Only 32% treated patients who had ≥ 4 lesions with only SRS [7].

Is the OS of patients who present with ≥ 4 brain metastases worse than that of those with 1-3? Few studies have evaluated the OS post-SRS treatment of patients with newly diagnosed 1-3 or ≥ 4 brain metastases without pre-SRS metastasectomy nor pre- or concurrent-to-SRS whole brain radiotherapy (WBRT) [8-13]. Bashir et al. was the only group to compare both arms simultaneously post-Gamma knife radiosurgery (GKR), again, as the sole treatment [14]. The present publication likewise compared both arms' OS, but used CyberKnife radiosurgery (CKRS) (Accuray, Sunnyvale, California) alone.

In the present paper, patients presenting with 1-3 and ≥ 4 brain metastases were evaluated for various parameters known to impact OS. The patient characteristics of age, gender, Eastern Cooperative Oncology Group (ECOG) performance score, number of extracranial disease (ECD) sites, ECD control or non-control at CKRS, alive status, and ECD versus central nervous system (CNS) disease as the cause of death (COD) were evaluated. Tumor characteristics, including histology, total tumor volume (TV) at initial CKRS treatment, and adjunct post-CKRS WBRT were documented. Brain magnetic resonance imaging (MRI) findings and date of brain metastasis local control (LC) (complete response (CR), partial response (PR), and stability) or local failure (LF), all with or without distal brain failure (DBF) were noted. The incidences of leptomeningeal disease (LMD) and radiation necrosis (RN) were also recorded.

In this paper, a method of predicting and facilitating imaging CR is presented. This system utilized CR and, in contrast, LF patients' complete blood counts with differentials as the source of both the absolute neutrophil count (ANC) which was divided by the absolute lymphocyte count (ALC), which is called the neutrophil-to-lymphocyte ratio (NLR). The NLR is a known prognosticator for OS in patients with primary carcinomas. Few papers, however, have utilized the NLR to analyze brain metastasis patients' post-SRS OS and imaging outcomes. Mitsuya et al. evaluated NLRs in pre-craniotomy patients having metastasectomies [15]. An NLR < five portended an OS of 14 months, while the OS was five months with an NLR ≥ five, Shaverdian et al. showed that in pre-SRS patients being treated for brain metastases, on multivariate analysis, a higher peripheral neutrophil percentage predicted poor initial brain MRI, LC, intracranial control of metastases, and OS [16]. A lower percentage of lymphocytes predicted an initial poor MRI and LC. It was not stated that ANCs, ALCs, or NLRs were used in their determinations, however.

As a background for how the ANCs and ALCs may play a role in controlling post-SRS brain metastases, peripheral blood lymphocytes (PBLs) have been found by Schondorff et al. to have similar cell types and cellular characteristics as tumor-infiltrating lymphocytes (TILs) at the site of primary breast and ovarian carcinomas [17]. This finding indicates that PBLs are the likely source of tumor TILs. Szeifert et al. performed histopathological and immunohistochemical evaluations of post-SRS controlled and uncontrolled brain metastases [18]. In the brain metastasis/brain parenchyma interface area and central necrotic region, TILs and tumor-associated neutrophils (TANs) were found, respectively, in controlled brain metastases, and these cells were sparse in these locations in less-well-controlled brain metastases. Thus, the TANs, which have as their likely source, neutrophils (ANCs), and TILs, lymphocytes (ALCs), appear to be involved in brain metastases control post-SRS. 

Thus, in the present paper, for the first time and continuing the preliminary work of Schondorff et al., ANC, ALC, and NLR values were determined at the time of CKRS for patients with CR and LF imaging outcomes. This preliminary study was carried out to ascertain if these values were predictive of CR versus LF imaging outcomes.

## Materials and methods

Study population

Charts of 574 Stanford University Medical Center (SUMC) patients post-CKRS treatment of brain metastases between 2009 and 2014 were reviewed retrospectively after approval by the Institutional Review Board (IRB) of protocol 26173. Excluded were patients who had undergone pre-CKRS metastasectomies or pre- or concurrent-to-CKRS WBRT and those who did not have post-CKRS brain magnetic resonance imaging (MRI) scans.

Data collection

Data for patient characteristics were collected and included each patient’s age, gender, ECOG score of 0, 1, or 2, number of ECD sites from 0 to ≥ 4, ECD control or non-control at CKRS, and alive status or COD due to ECD versus CNS disease.

The tumor characteristics of individual histological types, total TV at initial CKRS treatment, and whether the patients had undergone CKRS alone or with post-CKRS WBRT were collected. Prescribed doses (PDs) of CKRS and number of fractions delivered were noted.

Brain MRIs performed immediately pre- and at four-to-six-week intervals throughout post-CKRS treatment determined imaging outcome characteristics of treated brain metastases using neuroradiology reports and target measurements. Measurements were made employing the electronic caliper function of the Centricity picture archiving and communication systems (PACS) (General Electric Healthcare, Milwaukee, WI), which allows a spatial resolution to 0.1 mm. The response assessment in neuro-oncology brain metastases (RANO-BM) method had the following adaptations: cases were included that had ≥ five CKRS target lesions [19]. The sum (of the) longest diameter (SLD) of each CKRS-treated target lesion at the time of CKRS was compared with the SLD at the time of occurrence of CR (disappearance of all CKRS target lesions) without DBF (new brain metastases outside the treated TVs) or at concurrent CR, PR (30% SLD decrease), stable disease (neither PR nor LF), or LF (20% SLD increase) with DBF or final imaging. The time between CKRS and imaging outcome was calculated. Local control (LC) was CR, PR, or stable brain MRI outcomes. The durations from CKRS treatment date to time of occurrence of RN using brain MRI plus histological verification and imaging-determined-LMD were noted.

Preliminary prognostic marker determinations

Using the complete blood count (CBC) with differential when available at the time of CKRS treatment, the ANC and ALC counts were obtained. The ANC/ALC ratio (NLR) was calculated for each patient who had CR and LF imaging outcomes. 

Statistical analyses

Patients with brain metastases treated with CKRS alone were divided into two groups: those who had 1-3 brain metastases targeted at the time of initial CKRS presentation (group 1) versus patients who had ≥ 4 (group 2). Continuous characteristics were summarized as medians with interquartile range (IQR) and categorical characteristics as counts and percentages.

Homogeneity was assessed for patient characteristics (age, sex, ECOG score, number of ECD sites, and COD), tumor characteristics (histology, total tumor volume, post-CKRS WBRT status) and brain MRI responses between groups 1 and 2. For the latter analyses, the Chi-square test (or the Fisher’s exact test when the cell count was low) for categorical variables and the Mann-Whitney U test for continuous variables were used. Histological types included non-small cell lung carcinoma (NSCLC), breast, melanoma, renal cell carcinoma (RCC), and “other” carcinomas (bladder, gastric, colorectal, thyroid, ovarian, testicular carcinoma, or tongue and nasal squamous cell carcinoma).

The brain MRI outcomes, including LC and its components, versus LF were also assessed statistically for the number and percentage of each outcome. All patients were followed from CKRS until death or May 1, 2017 (end of the database). Kaplan-Meier estimates were computed by group (1-3 versus ≥ 4 brain metastases), and the OSs were compared using the logrank test. The association between the brain metastasis group and mortality was also assessed using uni- and multivariable Cox proportional hazards models, which adjusted for various patient and tumor characteristics. All statistical tests were performed at the 0.05 significance level, and analyses were implemented using the R-3.3 software [20].

## Results

Patient characteristics

Of 574 SUMC patients with brain metastases treated with CKRS during 2009-2014, 150 (26.1%) had such lesions treated with only CKRS. The median age for the 115 group 1 patients was 62, and it was 56 for the 35 group 2 patients (p = 0.124) (Table 1). There were 51 (44.3%) females and 64 (55.7%) males in group 1 and 20 (57.1%) and 15 (42.9%), respectively, in group 2 (p = 0.184). At CKRS treatment, group 1 patients predominantly had an ECOG performance status score of 1 (84 (73.0%)) as did group 2 patients (27 (77.1%)) (p = 0.045) and in group 1, 33 (28.7%) had one ECD site and group 2, two (12 (34.3%) (p = 0.473). The ECD in group 1 was uncontrolled in 83 (72.2%) and controlled in 32 (27.8%) and in group 2, in 29 (82.9%) and in six (17.1%) (p = 0.203), respectively. Eleven (9.6%) group 1 patients were alive at this study's conclusion and three (8.6%) in group 2. The COD for 85 (73.9%) group 1 patients was ECD and CNS in 19 (16.5%). For 21 (60.0%) group 2 patients, the COD was ECD and for 11 (31.4%), CNS (p = 0.155).

**Table 1:** Characteristics of Patients with 1-3 versus ≥ 4 CKRS-treated Brain Metastases * p-values were obtained using the Chi-square or Fisher’s exact test for categorical variables and the Mann-Whitney U test for continuous variables CKRS = CyberKnife radiosurgery; n = patient number; IQR = interquartile range; ECOG = Eastern Oncology Cooperative Group; ECD = extracranial disease; CNS = central nervous system

	Number of Brain Metastases			p-value*
	1-3 (n, 115)	≥ 4 (n, 35) (range 4-12)		(1-3 versus ≥ 4)
Age, years (median (IQR))	62.0 (53.5, 69.0)	56.0 (45.5, 66.5)		0.124
Sex (median (IQR))				0.184
Female	51 (44.3)	20 (57.1)		
Male	64 (55.7)	15 (42.9)		
ECOG (n (%))				0.045
0	19 (16.5)	1 (2.9)		
1	84 (73.0)	27 (77.1)		
2	12 (10.4)	7 (20.0)		
ECD - number of sites (n (%))				0.473
0	23 (20.0)	7 (20.0)		
1	33 (28.7)	6 (17.1)		
2	26 (22.6)	12 (34.3)		
3	18 (15.7)	7 (20.0)		
≥ 4	15 (13.0)	3 (8.6)		
ECD - status (n (%))				0.203
Uncontrolled	83 (72.2)	29 (82.9)		
Controlled	32 (27.8)	6 (17.1)		
Cause of Death (n (%))				0.155
Alive	11 (9.6)	3 (8.6)		
ECD	85 (73.9)	21 (60.0)		
CNS	19 (16.5)	11 (31.4)		

Tumor characteristics

Subgroups for the tumor characteristic of histology for group 1 were as follows: 63 (54.8%) had NSCLC, 18 (15.7%) breast carcinoma, 15 (13.0%) melanoma, 8 (7.0%) RCC, and 11 (9.6%) had “other” carcinoma types (Table 2). The tumor histology subgroups for group 2 included 25 (71.4%) who had NSCLC, 4 (11.4%), breast carcinoma, 4 (11.4%), melanoma and 1 (2.9%) each had RCC and an “other” carcinoma type as described in Table 2 (p = 0.528). The median total CKRS TV for group 1 was 1.08 ccs (IQR 0.30-3.33 ccs) and for group 2, 1.42 ccs (IQR 0.62-4.32 ccs) (p = 0.380). For group 1 patients, 101 (87.8%) versus 14 (12.2%) and for group 2 patients 24 (68.6%) versus 11 (31.4%) underwent CKRS without versus with post-CKRS WBRT, respectively (p = 0.007).

**Table 2:** Tumor Characteristics of Patients with 1-3 versus ≥ 4 CKRS-treated Brain Metastases * p-values were obtained from the Chi-square or Fisher’s exact test for categorical variables and from the Mann-Whitney U test for continuous variables. ** Other = 1-3 brain metastases: bladder (1), gastric (1), colorectal (1), thyroid (2), ovarian (1), testicular carcinoma (1), and tongue (3) and nasal (1) squamous cell carcinoma; ≥ 4 brain metastases: colorectal (1) CKRS = CyberKnife radiosurgery; n. = number of patients; NSCLC = non-small cell lung carcinoma; TV = tumor volume; IQR = interquartile range; WBRT = whole brain radiotherapy

	Number of Brain Metastases			p-value*
	1-3 (n, 115)	≥ 4 (n, 35) (range 4-12)		(1-3 versus ≥ 4)
Histology (n (%))				0.528
NSCLC	63 (54.8)	25 (71.4)		
Breast cancer	18 (15.7)	4 (11.4)		
Melanoma	15 (13.0)	4 (11.4)		
Renal cell carcinoma	8 (7.0)	1 (2.9)		
Other**	11 (9.6)	1 (2.9)		
TV (cc) (median (IQR))	1.08 (0.30, 3.33)	1.42 (0.62, 4.32)		0.380
WBRT–post-CKRS (n (%))				0.007
Without	101 (87.8)	24 (68.6)		
With	14 (12.2)	11 (31.4)		

Imaging outcomes

Pre- and post-CKRS brain MRIs were available for 115 patients in group 1 versus for 35 in group 2 of whom 93 (80.9%) versus 26 (74.3%) had brain metastasis LC (p = 0.400) (Table 3). Of patients having LC, for group 1 versus group 2: 32 (27.8%) versus 6 (17.1%) had CR (p = 0.203), 38 (33.0%) versus 18 (51.4%), PR (p = 0.049) and 23 (20.0%) versus 2 (5.7%) had stable imaging outcomes (p = 0.067). Local failure occurred in 22 (19.1%) of group 1 patients versus 9 (25.7%) of group 2 (p = 0.470). Distal brain failure was documented in 62 (53.9%) in group 1 and 21 (60.0%) in group 2 (p = 0.526). Eight (7.0%) group 1 patients developed LMD versus five (14.3%) in group 2 (p = 0.177) and five (4.3%) cases of RN occurred in group 1 patients alone (p = 0.591).

**Table 3:** Comparison of CKRS-treated Brain MRIs in Patients with 1-3 versus ≥ 4 Brain Metastases * p-values were obtained using the Chi-square or Fisher’s exact test. ** MRI and histologically documented CKRS = CyberKnife radiosurgery; No. = number; n = number; MRI = magnetic response imaging; DBF = distal brain failure; LMD = leptomeningeal disease; RN = radiation necrosis

		No. of Brain Metastases at 1stCKRS			P-value*
		1-3 (n, 115)	≥ 4 (n, 35)		
MRI Response (n (%))					
Local Control		93 (80.9)	26 (74.3)		0.400
	Complete Response	32 (27.8)	6 (17.1)		0.203
	Partial Response	38 (33.0)	18 (51.4)		0.049
	Stable	23 (20.0)	2 (5.7)		0.067
1-Year Local Control		82 (71.3)	24 (68.6)		
Local Failure		22 (19.1)	9 (25.7)		0.470
DBF		62 (53.9)	21 (60.0)		0.526
LMD		8 (7.0)	5 (14.3)		0.177
RN **		5 (4.3)	0 (00.0)		0.591

Overall survival

**Figure 1:**
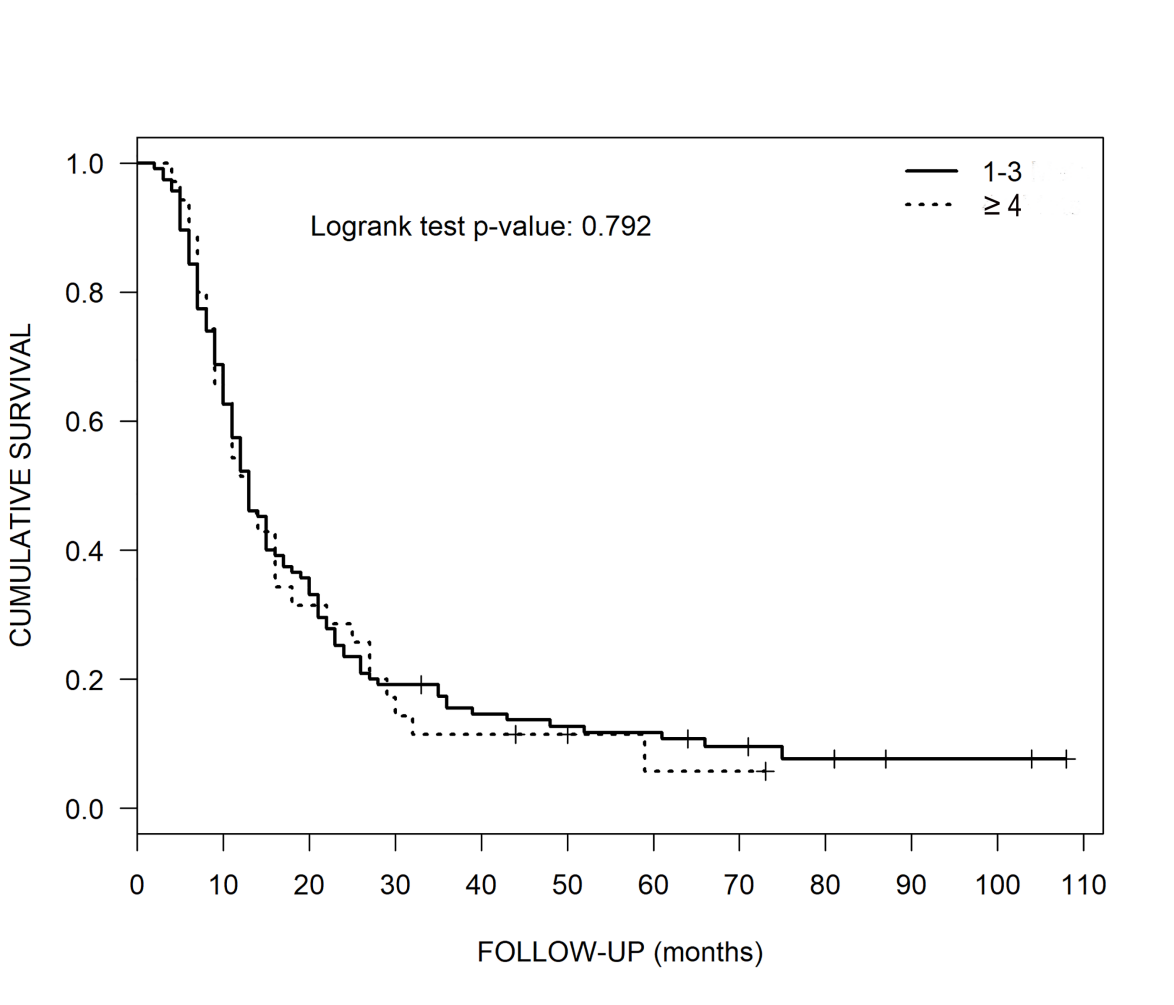
Overall Survival of Patients with 1-3 versus ≥ 4 Kaplan-Meier overall survival curves of patients with 1-3 versus ≥ 4 brain metastases treated with CyberKnife radiosurgery.

For 115 patients in group 1, the median OS was 13 months, 95% confidence interval (CI) (11-16) (Table 4, Figure 1). Thirty-five patients in group 2 also had a median OS of 13 months, 95% CI (10-22).

**Table 4:** Median OS and 95% CI Post-CKRS by No. of Brain Metastases* * The OSs in patients with 1-3 versus ≥ 4 brain metastases treated with CKRS were compared using the logrank test and uni- and multivariable Cox proportional hazards models. CKRS = CyberKnife radiosurgery; OS = overall survival; CI = confidence interval; No. = number; n = number; mos = months

	Patient No.	n Events	Median OS (mos)	95% CI
Number of Brain Metastases				
1-3	115	104	13	(11, 16)
≥ 4	35	32	13	(10, 22)

Tables 1-3 show that patients with 1-3 brain metastases were similar to those with ≥ 4 brain metastases with respect to all characteristics, except for ECOG (p = 0.045) (Table 1), WBRT post-CKRS treatment (p = 0.007) (Table 2) and imaging outcomes of PR (p = 0.049) and stable (p = 0.067) (Table 3).

Uni- and multivariable analyses

Using uni- and multivariable analyses, a poor ECOG score, greater ECD burden, and uncontrolled ECD status were significantly associated with a lower OS, with and without accounting for other covariates. Uni- and multivariable analyses confirmed that men were at significantly greater risk for a lower OS when gender was assessed alone, but not so in adjusted analyses. Also, when using a multivariate analysis, group status (1 versus 2) was not statistically significantly associated with OS after adjusting for various confounding factors (Table 5). 

**Table 5:** Uni- and Multivariable Cox Proportional Hazards Analyses of the Impact of Various Parameters on OS in CKRS-treated Patients with Brain Metastases (n = 150) * Other = bladder, gastric, colorectal, thyroid, ovarian, testicular carcinoma, tongue and nasal squamous cell carcinoma. OS = overall survival; CKRS = CyberKnife radiosurgery; n = number; HR = hazard ratio; CI = confidence interval; TV = tumor volume; NSCLC = non-small cell lung carcinoma; REF = reference; ECOG = Eastern Cooperative Oncology Group; ECD = extracranial disease; No. = number; WBRT = whole brain radiation therapy

	Univariable				Multivariable		
	HR	95% CI	P-value		HR	95% CI	P-value
≥ 4 Metastases (versus 1-3)	1.05	(0.71, 1.56)	0.803		0.94	(0.61, 1.47)	0.801
Male Sex	1.44	(1.02, 2.02)	0.038		1.13	(0.76, 1.67)	0.538
Age (years)	1.00	(0.99, 1.02)	0.553		1.01	(0.99, 1.02)	0.354
Total TV (cc)	1.04	(0.99, 1.08)	0.101		1.03	(0.98, 1.07)	0.204
Histology							
NSCLC	1.00	REF	REF		1.00	REF	REF
Breast cancer	0.52	(0.30, 0.91)	0.021		0.44	(0.23, 0.84)	0.013
Melanoma	1.30	(0.78, 2.17)	0.315		1.29	(0.72, 2.32)	0.398
Renal cell carcinoma	1.19	(0.60, 2.38)	0.615		0.64	(0.29, 1.44)	0.281
Other*	1.15	(0.61, 2.16)	0.666		0.82	(0.41, 1.64)	0.583
ECOG							
0	1.00	REF	REF		1.00	REF	REF
1	1.57	(0.94, 2.63)	0.087		1.84	(1.04, 3.24)	0.036
2	2.14	(1.09, 4.18)	0.027		2.75	(1.32, 5.73)	0.007
ECD – No. of Sites	1.15	(1.02, 1.30)	0.027		1.19	(1.01, 1.39)	0.039
Uncontrolled ECD	2.15	(1.41, 3.29)	<0.001		2.46	(1.54, 3.95)	<0.001
Post-CKRS WBRT	0.78	(0.50, 1.21)	0.266		0.66	(0.40, 1.08)	0.100

Prognostic marker analyses

At the time of CKRS treatment, 19 group 1 and 2 patients with CRs had a mean ANC of 5.88 K/µL and a mean ALC of 1.31 K/µL. Of these 19 CR patients, 13 (68%) had NLRs ≤ five also at CKRS (Table 6). At CKRS, 11 group 1 and 2 patients with LFs had a mean ANC of 5.22 K/µL and a mean ALC of 0.93 K/µL. Of these 11 LF patients, seven (64%) had NLRs > five. An NLR ≤ five and a higher ALC were associated with a CR outcome. Conversely, an NLR > five and a low ALC mean were associated with an LF outcome. 

**Table 6:** ANC, ALC and NLR Values at CKRS * K/µL ** mo = month(s) of administration (1st CKRS treatment day = time 0) ANC = absolute neutrophil count; ALC = absolute lymphocyte count; NLR = neutrophil-to-lymphoyte ratio (ANC/ALC); CKRS = CyberKnife radiosurgery; mg = milligram; NSCLC = non-small cell lung carcinoma; PMX = pemetrexed; MM = malignant melanoma; TC = testicular carcinoma; PEB = bleomycin, etoposide, cisplatin; RCC = renal cell carcinoma; DTIC = decarbazine; CRC = colorectal carcinoma

Histology	CKRS ANC*	CKRS ALC*	CKRS NLR	Steroids (mg)(mo)**	Chemo-, Immunotherapy
CR					
1-3 Brain Metastases			NLR ≤ 5		
Bladder	8.85	2.01	4.40	4 (0)	Gemzar, carboplatin
Breast	3.85	1.80	2.14	126 (0-6)	Gemzar, carboplatin
Breast	1.46	1.44	1.01	none	Herceptin
Breast	3.20	0.60	5.33	200 (0-3)	Taxol, Herceptin
NSCLC	4.96	1.00	4.96	89 (0-2)	Docetaxel
NSCLC	4.67	1.15	4.06	48 (0-16)	Tarceva
NSCLC	3.23	1.32	2.45	none	Tarceva
NSCLC	10.16	2.06	4.93	36 (0-5)	PMX, carboplatin, avastin
NSCLC	4.28	1.24	3.45	78 (0-12)	PMX, carboplatin, avastin
NSCLC	3.60	1.83	1.97	8 (0-3)	Taxol, carboplatin, avastin
NSCLC	4.90	1.60	3.06	88 (0-3)	Taxol, carboplatin, avastin
			NLR > 5		
MM	5.60	0.83	6.75	4 (0)	Ipilimumab
MM	2.10	0.33	6.36	8 (0)	Interferon-2b, Temodar
NSCLC	16.72	0.87	19.22	30 (1-9)	PMX, carboplatin
NSCLC	11.34	0.97	11.69	46 (9-6)	PMX, carboplatin
NSCLC	8.38	0.94	8.91	81 (0-4)	Gemzar, carboplatin
TC	8.01	0.90	8.90	214 (0-2)	PEB
≥ 4 Brain Metastases			NLR ≤ 5		
NSCLC	3.14	1.45	2.17	24 (0-3)	PMX, carboplatin
RCC	3.34	2.50	1.34	12 (0)	Nivolumab
Mean Values	5.88	1.31	5.43	64.47	-
LF					
1-3 Brain Metastases			NLR ≤ 5		
Breast	1.65	1.01	1.63	4 (0)	Herceptin, eribulin
RCC	2.55	0.50	5.10	4 (0)	Pazopanib then temsirolimus
			NLR > 5		
Breast	4.61	0.32	14.40	none	Herceptin, navelbine
MM	8.40	1.07	7.85	13 (0-2)	DTIC then ipilimumab
RCC	13.26	1.38	9.61	26.25 (0)	None
≥ 4 Brain Metastases			NLR ≤ 5		
NSCLC	3.41	1.55	2.20	60 (0-11)	PMX
NSCLC	3.35	1.33	2.52	none	Tarceva, dovitinib
			NLR > 5		
Breast	8.72	1.31	6.66	28 (01)	Xgeva
Breast	3.83	0.68	5.63	12 (0-2)	Lapatinib
NSCLC	3.24	0.40	8.10	43 (0-2)	Gemzar
CRC	4.39	0.64	6.86	20 (0)	Oxaliplatin, avastin, capecitabine
Mean Values	5.22	0.93	6.42	23.36	

## Discussion

The most common number of brain metastases at presentation is 1-3 [6]. A recent survey by Tsao et al., documenting predominantly radiation oncologists’ responses, showed that the maximum number of brain metastases they would treat with SRS was 3 [7]. Few publications have compared OSs after SRS treatment without pre-SRS metastasectomies nor pre-SRS or concurrent-with-SRS WBRT of patients presenting with 1-3 brain metastases versus those presenting with ≥ 4 to refute the latter management scheme.

Literature review

Overall survival: A PubMed literature review of publications that assessed OS after SRS without pre-SRS metastasectomies nor pre-SRS or concurrent WBRT in patients presenting with 1-3 versus ≥ 4 brain metastases was performed. The terms stereotactic, Gamma Knife (Elekta, Stockholm, Sweden), CyberKnife, linear accelerator (LINAC), radiosurgery, brain metastases, outcomes, survival, mortality, 1-3, and four and more were used. The search was limited to English language articles only, but not by date, age group, or type of publication. This review found that of seven investigators (after excluding the present paper), five evaluated OS for solely the 1-3 brain metastases group [8-12]. Conversely, only one group studied OS only in patients presenting with ≥ 4 brain metastases (Table 7) [13].

**Table 7:** Publications Evaluating Patients' OS with 1-3 and ≥ 4 Brain Metastases Using SRS Alone OS = overall survival; SRS = stereotactic radiosurgery; No. = number; mos = months; ECOG = Eastern Cooperative Oncology Group; KPS = Karnofsky Performance Status; RPA = recursive partitioning analysis; GKR = Gamma knife radiosurgery; LINAC = linear accelerator; CKRS = CyberKnife radiosurgery

	Brain Metastases, Patient No.				Brain Metastases No., Median OS (mos)	
Publication	1-3	≥ 4	ECOG, KPS, RPA	SRS	1-3	³ 4
Bashir et al. [14]	243	85	KPS 30 – 100	GKR	8	7.5
Schuttrumpf et al. [12]	189	N/A	RPA 1-3	LINAC	7.6	N/A
Murovic et al.	115	35	ECOG 0-2	CKRS	13	13
Brown et al. [9]	111	N/A	ECOG 0-2	Unspecified	10.4	N/A
Lutterbach et al. [11]	101	N/A	KPS ≥ 50	Elekta	7.6	N/A
Rades et al. [8]	95	N/A	RPA 1 – 2	LINAC, GKR	13	N/A
Ojerholm et al. [13]	N/A	38	RPA 2 – 3	GKR	N/A	6.7
El Gantery et al. [10]	18	N/A	KPS ≥ 70	LINAC	8	N/A

Thus, the five groups who evaluated the OS of patients with 1-3 brain metastases included Schuttrumpf et al., who used a LINAC system alone to treat 189 patients with 1-3 brain metastases and the median OS was 232 days (7.6 months) [12]. Brown et al. documented 111 patients with ECOG scores of 0-2 and ≤ three solely-SRS-treated brain metastases at presentation, who had a median OS of 10.4 months [9]. Lutterbach et al. presented the results of their review of 101 patients, also with only patients having ≤ three brain metastases treated with an Elekta alone. The latter group of patients had a median OS of 7.6 months [11]. Rades et al. stated that, in general, the outcome for patients with 1-3 brain metastases “appears to be better than that for patients with > 3 lesions” [8]. This group, however, analyzed the median OS of 95 patients, but also only those with 1-3 brain metastases, who had recursive partitioning analysis (RPA) scores of 1-2. They used linear accelerator-based SRS alone or Gamma knife radiosurgery (GKR) alone to attain a median OS of 13 months. El Ganterey et al. utilized a LINAC device alone to treat 18 patients with 1-3 brain metastases, who had a median OS of eight months [10].

The publication by Ojerholm et al. addressed OS in 38 patients who had only the higher number of brain metastases, i.e. ≥ 4 [13]. Their patients were RPA Class 2-3 and were treated with GKR alone. These patients attained an OS of 6.7 months.

Bashir et al. were the only authors, besides those of the present paper, to determine the median OS for patients with 1-3 brain metastases (eight months) and included a separate OS analysis of those with ≥ 4 (7.5 months) [14]. These authors, however, used GKR, again alone, rather than CKRS, as in the present study.

The present paper addresses the paucity of publications that simultaneously evaluate the median OSs of patients with both 1-3 and ≥ 4 lesions, who were treated with CKRS, without prior craniotomies for brain metastases resection or pre- or concurrent with CKRS WBRT. The mean OS for the five studies that evaluated the 1-3 arm was 9.3 months. The median OS was 6.7 months for patients with ≥ 4 brain metastases per a single study by Ojerholm et al., who evaluated this group. The difference between the mean OS for each of the two groups above of 9.3 months versus 6.7 may be due to the relatively small number of 38 patients in the ≥ 4 brain metastasis group versus the total of 514 patients who had 1-3 brain metastases at presentation in the other five studies.

For this paper’s 115 patients in group 1, the median OS was 13 months, while 35 patients in group 2 also had a median OS of 13 months (Table 7, Figure 1). The present paper’s results show longer OSs as compared to the similar study of Bashir et al. using GKR alone, the latter with a median OS of eight months for patients with 1-3 brain metastases and for those with ≥ 4, 7.5 months [14]. Bashir et al.’s patients’ KPS scores were 30-100, however, the present paper’s patients all had high ECOG scores of 0-2, which per Ma et al. are approximately equivalent to KPS scores of 60-100 [21]. This likely contributed to the difference in OSs between the two studies.

Statistical analyses

After adjusting for various confounding factors using a multivariable analysis, statistical analysis of the data from the present study showed that the number of brain metastases at the time of presentation is not significantly associated with OS, while the independent parameters, however, of uncontrolled ECD, greater number of ECD sites, and a lower ECOG score were, with and without accounting for other covariates. Men were at significantly great risk when gender was assessed alone, but, again, not in the adjusted analyses. The latter gender greater risk may be impacted in our study by the fact that the majority of patients had NSCLC as their histological diagnosis. Ferguson et al. documented a male preponderance of 478 (62%) versus 294 (38%) women in their study of gender-associated differences in patients with lung cancer [22]. Women with NSCLC had a better OS of 8.5 months versus 5.4 for men. The present paper’s group 1 NSCLC patients had a preponderance of 39 (62%) men versus 24 (38%) females, so this likely negatively impacted the median OS for this group 1 since women have better OSs with this disease. For the 25 NSCLC patients with ≥ 4 brain metastases in the present study, however, there was a predominance of 14 women (56%) versus 11 men (44%), which may have slightly positively skewed the OS of group 2 via the NSCLC category, though the impact of the latter was less due to the lower number of patients in group 2.

Local control rates

For Brown et al.'s 105 patients, equivalent to our group 1 patients, the three-month LC rate was 79 (75.3%) [9]. Rades et al.'s RPA Class 1 (35) and 2 (60) patients LC rates were similar at 59% and 71%, respectively [8]. El Gantery et al. achieved a one-year LC of 4 of 18 (22.2%) [10]. Of the present study's 115 group 1 patients with 1-3 brain metastases, 82 (71.3%) achieved a one-year LC and of 35 group 2 patients with ≥ 4 brain metastases, the one-year LC rate was 24 (68.6%).

Of 115 patients in the present study with 1-3 brain metastases at presentation, 93 (80.9%) had overall LC and of 35 patients who had ≥ 4 brain metastases, 26 (74.3%) achieved this. Our LCs for both groups may be even higher since some of the LFs likely were instances of pseudo-progression, which were categorized as LFs, rather than LCs.

Complications

Of 101 patients with 1-3 brain metastases treated with a LINAC system alone, Lutterbach et al. noted one incidence (1%) of “likely RN,” though this was not documented histologically due to the patient’s expiration [11]. Of 38 solely GKR-treated patients evaluated by Ojerholm et al., only one RN (3%) case occurred also [13]. In the present study, five (4.3%) instances of histologically documented RN were noted in group 1 patients only; our patient number of 150 was much higher than the numbers of the latter two studies.

Increasing CR outcomes using ALC and NLR values

Szeifert et al. have shown that TANs and TILs are both present in well-controlled SRS-treated brain metastases; these cells have pro- and anti-tumor effects, respectively, per Coffelt et al. [18,23]. These latter authors reported that systemic neutrophil depletion in mice with mammary breast cancers lead to a reduction in lung metastases. They also demonstrated in these mice that the mammary tumors produce interleukin (IL)1β, which induces γδ T cells to produce IL17. The increased systemic IL17 level upregulates granulocyte-colony stimulating factor (G-CSF), which drives the systemic expansion and alteration of neutrophil phenotypes. These altered neutrophils produce inducible nitric oxide synthase (iNOS), which suppresses an anti-tumor cluster of differentiation 8 (CD8) T cell activity (CD8+ T cells). This results in subsequent increased chances of metastasis formation in distant organs. Thus, this cascade of events likely occurs in the post-SRS brain metastases. This cascade may actually contribute to the control of post-SRS brain metastases by TILs. The number of TILs may be reduced by the TANs so as not to cause overstimulation of the TILs, which would cause pseudo-progression.

Stereotactic radiosurgery also stimulates TILs per Szeifert et al., and this may occur by SRS blood-brain barrier damage. This damage allows lymphoid infiltration into the perivascular space of the brain surrounding the tumor. They noted that SRS causes brain metastasis cells to act as an increased activation stimulus for systemic T cells, resulting in the abscopal effect [18]. 

ALC mean values in CR versus LF patients:* *The CR patients’ CKRS ALC mean was 1.31 K/µL, while the LF patients’ mean value was 0.93 K/µL The CR patients thus likely have a higher reservoir of lymphocytes than the LF patients for brain metastases control by TILs post-SRS. This postulation would correlate with Szeifert et al.'s findings of more TILs surrounding controlled brain metastases and the converse in the uncontrolled brain metastases described above.

NLR mean values in CR versus LF patients: A preliminary analysis of NLRs ≤ 5 and > 5 at the time of CKRS treatment for CR and LF imaging outcome patients was carried out in the present paper as one of the first in the literature. Of 19 groups 1 and 2 CR imaging outcome patients, the majority or 13 (68%) had NLRs ≤ 5. Of 11 groups 1 and 2 LF imaging outcome patients, the majority or seven  (64%) had NLRs > five. Thus, NLRs ≤ five were associated with a higher chance of a CR imaging outcome. This reflects the higher mean ANC numerator value of 5.88 K/µL in the CR patients versus 5.22 K/µL in the LF patients and the higher ALC denominator value of 1.31 K/µL for the CR patients versus 0.93 K/µL for the LF patients, obtained from individual NLR calculations for each patient.

The mean steroid administration for the interval between CKRS and CR outcome was 64 mg, while for the LF patients, 23 mg. Cook et al. evaluated peripheral blood mononuclear cells after decadron administration and found that the regulatory T cells underwent significant increase and proliferation [24]. Tanaka et al. stated that the regulatory T cells suppress the anti-tumor immune response [25]. Alternatively, glucocorticoids also enhance the activity of macrophages and induce tolerogenic dendritic cells, exerting a potent anti-inflammatory effect [26-27]. The latter is possibly the reason that the CR patients who received larger amounts of steroids than did the LF patients did so well regarding their imaging outcomes since Szeifert et al. showed that macrophages were found with plasmocytes at SRS-treated brain metastases, which were controlled [18].

Techniques to increase CR outcomes: The number of patients with CR could be increased if the patients had monitoring of ANC, ALC, and NLR values during the pre- and post-CKRS time intervals. These values would be used to determine and possibly to adjust the chemo- and immunotherapies and other agents, such as steroids, which patients undergoing CKRS for brain metastases receive, the latter with possibly deleterious effects on ALCs and possibly ANCs. The steroid beneficial effects on the patients' ANC, ALC, and NLR levels needs further study with a larger number of CKRS-treated brain metastasis patients. On the other hand, the patients received adjunctive therapies, such as Gemzar and other agents (Table 6), all of which may lower the ALCs, but, in turn, also have immuno-stimulatory properties [28]. Thus, the ANC, ALC, and NLR evaluation appears to be promising and needs to be studied further in more patients with CKRS-treated brain metastases to gain more insight into how the CR outcomes of CKRS can be influenced and increased.

## Conclusions

The median OS was 13 months in patients who presented with 1-3 brain metastases treated with CKRS and it was also 13 months for those with ≥ 4 brain metastases treated with CKRS. The independent parameters of uncontrolled ECD, greater number of ECD sites, and lower ECOG scores were significantly associated with a greater mortality risk with and without accounting for other covariates. Men were at significantly greater risk when gender was assessed alone, but not in the adjusted analyses. This publication represents one of the only studies to use CKRS alone to evaluate both groups of patients with 1-3 and 4 or more brain metastases. An LC rate in 150 combined group 1 and group 2 patients of 119 (79%) was achieved and, of these, a 25% CR imaging outcome occurred, all with minimal toxicity. Of 19 groups 1 and 2 patients with CRs, 13 (68%) had NLRs ≤ five and all 19 had a mean ALC of 1.31 K/µL at the start of the CKRS treatment. Of 11 group 1 and group 2 patients with LFs, seven (64%) had NLRs > five and 11, a mean ALC of 0.93 K/µL. An NLR ≤ five and a higher ALC was associated with a CR outcome. An NLR > five and low ALC mean predicted an LF outcome.
